# Curcumin-Loaded Silica Nanoparticles: Applications in Infectious Disease and Food Industry

**DOI:** 10.3390/nano12162848

**Published:** 2022-08-18

**Authors:** Solmaz Maleki Dizaj, Simin Sharifi, Fatemeh Tavakoli, Yaseen Hussain, Haleh Forouhandeh, Seyed Mahdi Hosseiniyan Khatibi, Mohammad Yousef Memar, Mina Yekani, Haroon Khan, Khang Wen Goh, Long Chiau Ming

**Affiliations:** 1 Department of Dental Biomaterials, Faculty of Dentistry, Tabriz University of Medical Sciences, Tabriz 5165665931, Iran; 2 Dental and Periodontal Research Center, Tabriz University of Medical Sciences, Tabriz 5165665931, Iran; 3 Lab of Controlled Release and Drug Delivery System, College of Pharmaceutical Sciences, Soochow University, Suzhou 215123, China; 4 Molecular Medicine Research Center, Biomedicine Institute, Tabriz University of Medical Sciences, Tabriz 5165665931, Iran; 5 Kidney Research Center, Tabriz University of Medical Sciences, Tabriz 5165665931, Iran; 6 Infectious and Tropical Diseases Research Center, Tabriz University of Medical Sciences, Tabriz 5165665931, Iran; 7 Department of Microbiology, Faculty of Medicine, Kashan University of Medical Sciences, Kashan 8715988141, Iran; 8 Student Research Committee, Kashan University of Medical Sciences, Kashan 8715988141, Iran; 9 Department of Pharmacy, Abdul Wali Khan University, Mardan 23200, Pakistan; 10 Faculty of Data Science and Information Technology, INTI International University, Nilai 78100, Malaysia; 11 PAP Rashidah Sa’adatul Bolkiah Institute of Health Sciences, Universiti Brunei Darussalam, Bandar Seri Begawan BE 1410, Brunei

**Keywords:** phytochemicals, mesoporous silica nanoparticles, drug delivery, bacteria, infections, food packaging

## Abstract

Curcumin has multiple properties that are used to cure different diseases such as cancer, infections, inflammatory, arthritic disease, etc. Despite having many effects, the inherent physicochemical properties—such as poor water solubility, chemical instability, low bioavailability, photodegradation, fast metabolism, and short half-life—of curcumin’s derivatives have limited its medical importance. Recently, unprecedented advances in biomedical nanotechnology have led to the development of nanomaterial-based drug delivery systems in the treatment of diseases and diagnostic goals that simultaneously enhance therapeutic outcomes and avoid side effects. Mesoporous silica nanoparticles (MSNs) are promising drug delivery systems for more effective and safer treatment of several diseases, such as infections, cancers, and osteoporosis. Achieving a high drug loading in MSNs is critical to the success of this type of treatment. Their notable inherent properties—such as adjustable size and porosity, high pore volume, large surface area, functionality of versatile surfaces, as well as biocompatibility—have prompted extraordinary research on MSNs as multi-purpose delivery platforms. In this review, we focused on curcumin-loaded silica nanoparticles and their effects on the diagnosis and treatment of infections as well as their use in food packaging.

## 1. Introduction

In the past few years, several investigations have focused on applying mesoporous silica-based platforms as efficient nanocarriers [1]. There are some favorite properties of using mesoporous silica nanoparticles (MSNs) as a nanocarrier, including having a large number of pores, wide surface area, and adjustable morphology of pore structures [2]. Inorganic silica features, (e.g., surface, size, and topology) can be changed to create separate interactions with different kinds of biological systems. Therefore, mesoporous silica, microporous crystalline titanosilicates, amorphous silica, and zeolites have been extensively utilized for biomedical purposes [3].

In general, MSNs have more long-term release in comparison with organic nanoparticles, because prodrugs can be trapped inside their nano-pores. However, if pro-drugs were trapped in organic nanoparticles, the organic substance would rapidly degenerate; because of the quick pro-drug release [4]. The nanotechnology based on MSNs is sufficiently mature to be expanded to thousands of prodrugs that have not been considered in clinical use yet [5].

The promising properties of mesoporous inorganic materials, particularly MSNs, are easily adapted to incorporate and interact efficiently with a range of low-solubility medications and biomolecules [6]. Therefore, it is clear that we can see a rise in interest in this field. One of the major challenges of MSNs characteristics is deep concerns about their pharmacokinetic and immunological properties which have been studied to be surpassed to realize their clinical potential [7].

Through the functionalization of silica nanoparticles with different coating agents, the loading can be more controlled. As untreated silica nanoparticles have been known to be highly negatively charged and have stable hydrodynamic sizes in a wide pH range, by controlling the surface charge of colloidal silica nanoparticles, a positively charged surface can be formed. Many coating agents—such as amine-containing molecules, multivalent metal cation, or amino acids—can be applied to positively treat the silica nanoparticles. Moreover, molecules with chelating sites have shown high affinity with the silica surface to produce gel-like networks. Amino acid coatings also can be resulted stable silica colloids [8]. MSNs have a greater ratio of surface to volume than bare particles, which makes them a good carrier for drug loading [9].

Clinical medicine has many medicinal products for therapeutic applications, and with a greater understanding of the molecular mechanisms of disease, this list is growing rapidly. Several herbal agents have low water solubility, and this is generally related to low oral bioavailability [10]. For developing new therapeutics, the most important thing to do is to design an appropriate pharmaceutical formulation for delivery.

One active substance that is found in the roots of the *Curcuma longa* plant is curcumin. It is composed of small natural molecular weight yellow-orange polyphenol, which has been used in wound healing, diabetes, and cardiovascular disease due to its outstanding antibacterial, anti-inflammatory, and antitumor activities [11,12,13,14,15].

A hopeful methodology to overcome systemic toxicity and low bioavailability is the application of nanocarriers, such as liposomes, polymeric nanoparticles, micelles, and dendrimers [16,17,18]. The application of such nanocarriers possesses numerous benefits compared to systemic application, it can modify the drug’s pharmacokinetics, and it may be improve drug delivery to target locations [19,20].

Besides proper surface properties, MSNs have good biocompatibility, easy surface modification, controllable size, etc., making them exceptional candidates for different medical applications [21,22,23]. Due to these exceptional features, the quantity of investigations on MSNs has increased. Large specific areas of surface and volume of pores make MSNs appropriate reservoirs for loading diagnostic/therapeutic agents. MSNs can protect their loaded agents from premature release and subsequent undesirable degradation in the stomach and intestines before they reach their target site [24,25].

This is the first review to consider MSNs as the carrier for curcumin for the diagnosis and treatment of infections, as well as considering their applications in the food industry. The necessity for such a review is a consequence of rapid progress in this field. This review may help investigators accelerate studies and development of this essential field of nanomedicine and, eventually, industrial and clinical applications.

## 2. Curcumin

Plants are important natural resources for the preparation of medicinal products for various therapeutic purposes [26,27,28,29]. Curcumin as a main phytochemical polyphenol has been revealed to target multiple signaling molecules. It also indicates activity at the cellular level, serving its numerous health profits [30,31,32]. Curcumin is known in many coteries in diverse forms for multiple potential health properties. It is obtainable in numerous procedures—including capsules, tablets, ointments, energy drinks, soaps, and cosmetics [30,33]. Curcuminoids have been determined by the US Food and Drug Administration (FDA) as “Generally Recognized As Safe” (GRAS), and proper safety outlines have been revealed by clinical trials, even at doses between 4000 and 8000 mg/day [34] and of doses up to 12,000 mg/day of 95% concentration of three curcuminoids: curcumin, bisdemethoxycurcumin, and demethoxycurcumin [35,36]. Table 1 shows the main characteristics of curcumin.

## 3. Silica Nanoparticles

Silica nanoparticles (SiNPs)—with vital benefits such as large surface area, simple functionalization, and biocompatibility—are one of the most frequently applied nanoparticles in drug delivery uses. A porous model, mesoporous silica nanoparticles (MSN), also shows additional features such as tunable pore size and volume, leading to high drug loading volume [43,44]. The main methods for preparing silica nanoparticles include flame synthesis, wet chemical synthesis method, precipitation method, acid treatment, reverse microemulsions, hydrothermal technique, and extensively used sol–gel procedure. The sol–gel method has the advantage to control the particle size, size distribution, and morphology of particles comparing the other mentioned methods. It leads to nanoparticles with narrow size distribution as compared to the wet chemical synthesis method. Both methods have demonstrated a long reaction time [44,45,46,47].

Table 2 summarizes the methods to prepare the silica nanoparticles. It specifies that the main basis compounds for SiO_2_ used in the production of the nanoscale SiO_2_ include organo silicates, silanes, chlorosilanes, tetraethylorthosilane, and waste ash from biomass.

There are different kinds of MSNs with different properties and applications [53]. Table 3 shows different types of MSNs. However, MSNs are talented drug delivery systems, as their loading capacity can be altered according to the physicochemical possessions of the drug to gain the desired results.

## 4. MSNs for Co-Delivery of Curcumin with Antibiotics

First, the ability of MSNs to inter the bacteria and biofilm has been illustrated, then the challenge is in fighting antibacterial resistance by designing nanosystems with improved antimicrobial effectiveness through combining different antimicrobial components in the same nanoplatforms. For this purpose, various studies have identified innovative strategies based on shared delivery of antibiotics or combining antibiotics and antimicrobial metal nanoparticles or ions [66,67].

Concerning the first approach, the easiest way is to load different antibiotic molecules into the MSNs’ pores at the same time. The co-loading of agents with different—and sometimes conflicting—physicochemical properties in 26-side a single MSN requires development of compartmentalization approaches. One of the interesting methods for co-delivery of therapeutic agents of different chemicals is the use of MSNs with asymmetric structure and anisotropic geometry and two compartments that can load drugs of different natures in separate storage spaces. Song et al. decorated silver nanoparticles in mesoporous silica of SBA-15 covered by melanin-like polydopamine (PDA) as carriers. During this time, the constructed mesopores were charged with curcumin (CCM) by their noncovalent connections with coatings of PDA. The acquired CCM@SBA-15/PDA/Ag compounds were characterized physiochemically and demonstrated low hemolytic action and positive biocompatibility [68]. Realizing the dual-stimuli-responsive (ROS and pH) from silver and/or curcumin nanoparticles from the composites of CCM@SBA-15/PDA/Ag was carried out to decrease the side effects of the drug leakage of non-controlled due to physiological circumstances. Moreover, in comparison to that of CCM@SBA-15/PDA and SBA-15/PDA/Ag, CCM@SBA-15/PDA/Ag combination displayed a long-lasting inhibitive effect on the growth of *S. aureus* (24 h) and *E. coli* (72 h), attributed to the increased impact of the bactericide of curcumin and silver nanoparticles [68].

Asymmetric MSNs with dual-compartments and anisotropic geometry are very desirable for charging and releasing dual drugs into separated storage areas. In a study, an asymmetric lollipop-like mesoporous silica nanoparticle Fe_3_O_4_@SiO_2_&EPMO (EPMO = ethane bridged periodic mesoporous organosilica) was extended effectively through an anisotropic epitaxial growth approach. The absorbance measurement at 600 nm evaluated the antibacterial effects of Fe_3_O_4_@SiO_2_-GS&EPMO-Cur. *S. aureus* and *E. coli* were selected as bacterial models. At the same concentration, Fe_3_O_4_@SiO_2_&EPMO-Cur and Fe_3_O_4_@SiO_2_-GS& EPMO-Cur have a better antimicrobial effect than curcumin, because of the enhanced solubility of curcumin loaded with the asymmetric lollipop-like nanoparticles. The rising concentration of curcumin, Fe_3_O_4_@SiO_2_&EPMO-Cur, and Fe_3_O_4_@SiO_2_-GS&EPMO-Cur caused the viability of *E. coli* and *S. aureus* to decrease considerably. In addition, Fe_3_O_4_@SiO_2_-GS&EPMO-Cur shows that it has much more capacity for inhibiting bacteria than Fe_3_O_4_@SiO_2_&EPMO-Cur in the same concentration. This is due to the synergic effect of curcumin and gentamicin concurrently loaded into the asymmetric lollipop-like nanoparticles [69].

## 5. Antibacterial Effect of Curcumin-Loaded MSNs

The antibacterial activity of curcumin on Gram-negative and Gram-positive species has been demonstrated. Cheng and coworkers designed novel core–shell magnetic MSNs. Curcumin and gentamicin were separately loaded into the hydrophobic and hydrophilic spaces to provide dual anticancer and antibacterial nanosystems [69].

A trio-hybrid nanocomposite of MSNs including copper, loaded with curcumin and decorated with Ag nanoparticles, represented the photokilling of *E. coli* [70]. The nanocomposite displayed a positive surface load, and Ag attendance raised the production of ROS. After irradiation, the trio-hybrids with curcumin at 1.5 µM diminished the viability of bacteria by 5 and 4 log in comparison with free curcumin and nanocomposite without curcumin, respectively. At 3 µM of curcumin, the bacterial cells were destroyed by the trio-hybrids. Nanocomposite-mediated aPDT bacterial cell damage is shown in SEM images [70]. A nanocomposite film of chitosan and curcumin-loaded MSNs was prepared and indicated an antibacterial effect against *E. coli* and *S. aureus*, which was evaluated by the disk diffusion test [71]. It has been seen that the inhibitory effect on *S. aureus* was more than *E. coli*; however, the antibacterial activity of chitosan with curcumin was more than the nanocomposite film [71].

**Figure 1:**
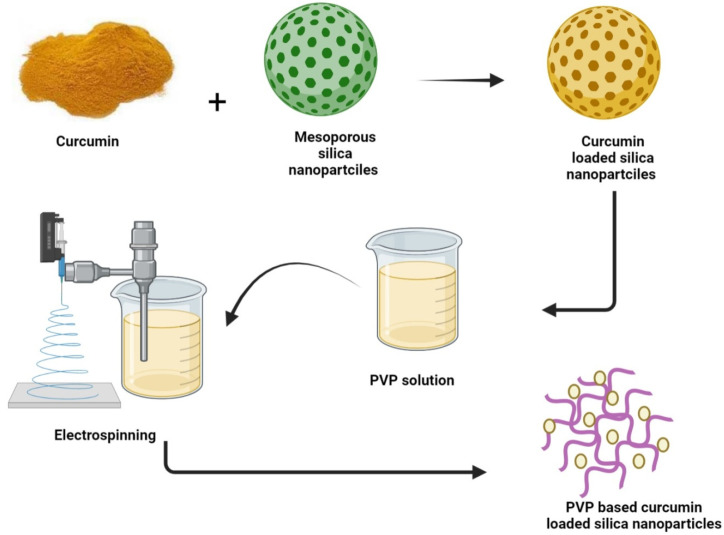
Fabrication of curcumin-loaded PVP based silica mesoporous nanofibers mat.

To combine the benefits of polyvinylpyrrolidone (PVP) with MSNs, Li et al. have designed a hybrid hemostatic organic–inorganic material using electrospinning by integrating the curcumin-loaded MSNs (CCM-MSNs) into PVP nanofiber mats (Figure 1). Furthermore, increased antibacterial activities against methicillin-resistant *S. aureus* (MRSA) were represented by hybrid nanofiber mats in vitro [72].

Curcumin is loaded into already prepared mesoporous silica nanoparticles and the resultant complex is introduced into a PVP (polyvinylpyrrolidone) solution. Electrospinning of the admixture leads to the drug arrangement into suitable positions of the polymer results in the production of hybrid PVP-based curcumin-loaded mesoporous silica nanofibers mat.

Kuthati et al. prepared copper-loaded MSNs (Cu-MSNs) with immobilized silver nanoparticles (SNPs) for applying photodynamic inactivation (PDI) to drug-resistant *E. coli*. SNPs were decorated over the surfaces of Cu-MSNs by coordination of silver ions on diamine-functionalized Cu-MSNs and with formalin further reduced to silver nanoparticles. The ability of silver in sensitizing *E. coli* as a specific phototherapeutic agent in vitro was shown, so it can expand the phototherapeutic spectrum of curcumin. After external decoration with silver nanoparticles, and following loading of curcumin, the mesoporous structure of Cu-MSNs remains intact. Various physical characterization techniques have confirmed the synthesis and successful assembly of the functional nanomaterials. Under light irradiation, curcumin may produce large quantities of ROS, which may enhance the kinetics of silver ion release for antimicrobial activity. Cu-MSNs positive-laden modified surfaces support antimicrobial response by electrostatic attractions to negative-load bacterial cell membranes.

**Figure 2:**
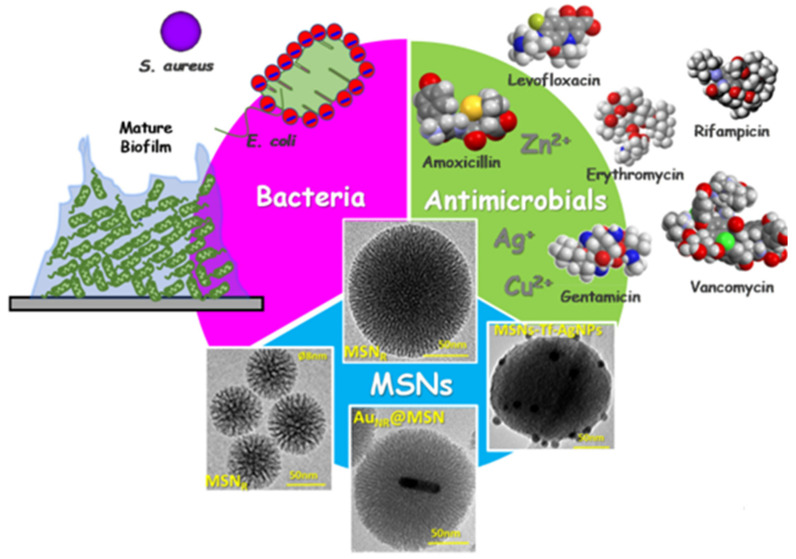
Schematic depiction of the three main actors starring in the innovative scientific approaches developed by Vallet-Regí et al. in the design and engineering of nanoantibiotics for the treatment of infections: bacteria, antimicrobial agents, and MSNs. MSNR: Radial mesoporous silica nanoparticles; AuNR@MSN: gold nanorods@mesoporous silica nanoparticles; MSN-Tf-AgNPs: mesoporous silica nanoparticles decorated with transferrin and silver nanoparticles. Reprinted from ref. [66].

A synergic mechanism for transferring energy of the absorbed light from SNP to curcumin may activate the antibacterial effect of the prepared nanocomposites [70]. Vallet-Regí and co-workers—who were working on bone infections—encountered a challenge in designing and developing advanced nano-antibiotics (Figure 2) [66].

First, bacteria that are present in infections of bone-implant (both in the planktonic and biofilm forms) should be locally destroyed in situ to prevent the need for surgical procedures for removing and replacing prostheses that were infected by bacteria. In contrast, in the infected area, antimicrobials—which may contain only antibiotics or the combination of metal ions and antibiotics—must be treated with small, high-specificity doses to enhance their efficiency and decrease dangerous side effects in healthy cells, tissues, and organs.

This aim can be achieved via nanocarriers capable of charging, protecting, and transporting antimicrobial agents to the target (free bacterial and biofilm form). Upon arrival, the nanocarriers gradually release antimicrobial agents in response to specific external or internal stimuli. Among nanosystems, MSNs came as the third item that meets all those demands. In this way, innovative and advanced technologies are used in the design and development of advanced targeted stimuli-responsive MSNs-based nanocarriers for antimicrobials administration.

## 6. Wound Healing Effect

It has been demonstrated that curcumin can tweak physiological and molecular occasions included in the inflammatory and proliferative stages of the wound healing process [73]. Curcumin has appeared to have substantial wound healing properties. It can influence several stages of the natural wound healing process to hurry this process. Poor pharmacokinetics of curcumin repress its potential. Curcumin nanoformulation possibly increases wound healing by preventing the inflammatory response, inducing angiogenesis, containing fibroblast proliferation, and promoting re-epithelialization and collagen synthesis. Hence, the nanoformulations of curcumin exhibit an efficient wound healing potential. Hamam et al. loaded curcumin onto mesoporous silica particles and explored their wound-healing impact in vivo [74]. Wistar rats were randomly divided into two groups: the control group was treated with standard drug sulfadiazine topically and the second group was treated with a 1% curcumin formulation with application twice a day. The excision diameter was observed on days 3, 6, 9, 12, 15, 18, and 21 of treatment. Moreover, three rats per group were selected on days 7, 14, and 21. For histological examination, cross sections were taken from selected rats from the excise lesion area to evaluate inflammation, angiogenesis, the proliferation of fibroblasts, reepithelization, and the attendance of collagen [74]. Curcumin nanoformulation did not have a significant effect on wound contraction in the treated group compared to the control group (*p* > 0.05). In each group, inflammatory responses impressively decreased by day 21 of treatment, the procedure of angiogenesis was nearly complete by day 7, wound reepithelization with a high degree was accomplished by day 21, and fibroblast proliferation significantly grew by day 14, without significant contrasts between the groups. Through day 21, the collagen level substantially extended in rats that had been treated with the curcumin nanoformulation as compared with the control group.

Mirzahosseinipour et al. studied the antibacterial photodynamic impact of curcumin silica nanoparticles and free curcumin as photosensitizer agents on planktonic and biofilm forms of *S. aureus* and *P. aeruginosa*. The photodynamic therapy (aPDT) effect on the planktonic and biofilm forms of bacteria was investigated at 465 nm.

One of the major issues in public health is the multidrug resistance of pathogenic bacteria. Subsequently, finding new techniques for fighting multidrug-resistant bacteria has become increasingly urgent. One forward-looking method is antimicrobial aPDT, which includes utilizing photosensitizer and nontoxic dyes, which are stimulated by visible light and produce oxygen-free radicals. In one study, researchers analyzed the antibacterial photodynamic activity of curcumin silica nanoparticles and free curcumin as photosensitizers on planktonic and biofilm forms of *S. aureus* and *P. aeruginosa*. It was concluded that if they use curcumin in the form of curcumin-silica nanoparticles, there would be a decrease in the amount of producing bacterial biofilm and the number of bacteria in planktonic states. Curcumin-silica nanoparticles displayed a wound healing effect in the scratch assay in vitro [75]. Xi et al. prepared a multifunctional elastomeric poly (l-lactic acid)-poly (citrate siloxane)-curcumin@polydopamine hybrid nanofibrous scaffold (indicated as PPCP matrix) for treating infections of tumors and wounds. The PPCP matrix exhibited multifunctional properties—such as photothermal, anticancer, anti-inflammatory, antibacterial, antioxidative, and angiogenesis activities. The polydopamine/curcumin displayed an amazing near-infrared photothermal/tumor toxicity, which supported PPCP for synergistic treatment of skin cancer with antibacterial properties. In addition, the PPCP nanofiber matrix hastened skin wound healing by improving early angiogenesis in normal and bacteria-infected mice.

Multifunctional bioactive PPCP nanofibers matrix offers a competitive technique for wound healing induced by skin cancer and bacterial infections [76].

Rathinavel et al. produced a composite nanoscaffold that has potential in wound healing therapeutics and contains curcumin, SBA-15 (Santa Barbara Amorphous), and amine-functionalized SBA-15 polycaprolactone (PCL). The excessive biocompatibility and cell adhesion of amine-functionalized SBA-15 and the extensively investigated antimicrobial effects of curcumin included advantages for wound healing. Antibacterial studies carried out on *Bacillus subtilis* and *E. coli* strains displayed an enhanced inhibition area. Research on Wistar rats via engineered nanofibers included with amine-functionalized SBA-15 and curcumin confirmed 99% scar-less wound recovery in 21 days.

The results of Masson’s tri-chrome and hematoxylin–eosin staining displayed tissue re-epithelialization, formation of granulation tissue, and collagen deposition. The fabricated nanoscaffold could be an efficient system for wound healing [77]. In another study, Rathinavel et al. designed and manufactured the polyvinyl alcohol (PVA) Santa Barbara Amorphous(SBA)-15 with curcumin, which can be utilized as a biomimetic scaffold for tissue engineering of skin.

The results of cell adhesion, MTT assay, and live/dead assay indicate that the nanoscaffold had a higher rate of biocompatibility, cytocompatibility, proliferation, and cell migration without toxicity on cells. The synthesized nanofiber forms a powerful nanomaterial for the treatment of skin wounds.

In the end, the results of in vivo study showed that SBA-15-incorporated PVA nanofiber with curcumin appeared to possess effective wound healing effects [78].

## 7. Antiviral Effect

Several investigations have been performed to study the antiviral potential of the herbal compound due to the lack of prevention and treatment options for numerous viral infections [79,80]. The curcumin showed antiviral effects against different viruses such as influenza viruses, hepatitis viruses, and chikungunya virus (CHIKV); or emerging arboviruses such as Zika virus (ZIKV) [81]. Interestingly, curcumin decreases the spread of sexually transmitted diseases such as human papillomavirus (HPV), human immunodeficiency virus (HIV), and herpes simplex virus 2 (HSV-2) [82,83,84]. Mesoporous material made from an iron–phenanthroline nanocomplex was utilized to encapsulate curcumin (NCIP) with antiretroviral effect against HIV-1 human microglial cells [85]. Immunofluorescence staining displayed that NCIP decreased HIV-p24 expression by 41%, while free curcumin decreased by 24%, which showed suppression of HIV replication. NCIP showed an anti-inflammatory effect with a decrease in interleukin-8 (IL-8), nitric oxide, and TNF-α expression. Antioxidant activity was also observed, as NCIP decreased expression of heme oxygenase and increased expression of catalase [85].

**Figure 3:**
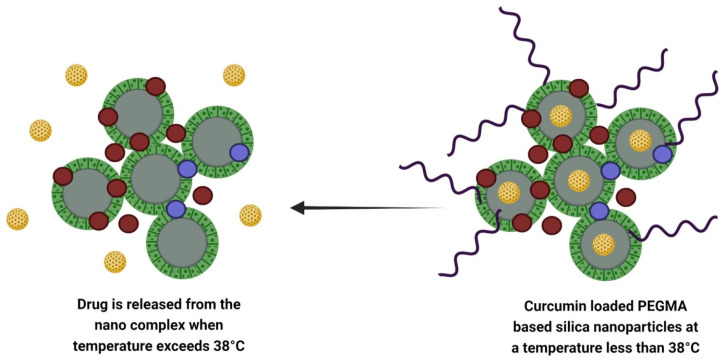
Design of curcumin-loaded temperature controlled dual imaging drug carrier system.

Lo et al. designed a temperature-controlled and dual imaging drug carrier that prevents Zika virus infection when loaded on curcumin (Figure 3). High fever in Zika infection is used as the basis to design a carrier that controls the release of drugs at a suitable temperature and which is sensitive to rising body temperature to release the drug if symptoms occur. To raise the biocompatibility and solubility of the curcumin, it was loaded into MSNs doped with PEGMA—temperature-responsive polymers and phosphorescent metal ions—i.e., Gd^3+^ and Eu^3+^. The experimental work was divided into two parts. Firstly, MSNs were synthesized with a temperature-controlled release feature and dual-imaging function with final loading through curcumin. Once formed, the dual imaging function (MSN-EuGd) can be achieved by calcination for pattern removal. Curcumin was loaded after surface modification. Secondly, curcumin-loaded mesoporous silica nanoparticle composite was compared with free curcumin for antivirus capability. Finally, the drug carrier with temperature-controlled features and dual imaging function was synthesized for inhibition of the Zika virus [86].

The PEGMA surface-modified EuGd-based mesoporous silica nanoparticles act as a temperature-controlled gatekeeper. When the temperature rises above 39 °C, the PEGMA turns into a coil that leads to the opening of the gate. Then, curcumin loads into the MSN pores. On another hand, when the temperature drops below 39 °C, the PEGMA becomes globular in shape over drug-loaded nanoparticles and closes the gate [86]. 

## 8. Application in Food Packaging

Recently, packaging materials with advanced technology have become a topic of great scientific and practical interest [87,88]. Formal regulations and public tendencies encourage the exploration of biodegradable substitutes for fossil fuel packaging, the extent of which causes widespread damage to the environment. Furthermore, modern packaging materials are predicted to out-perform passive packaging by protecting the enclosed product and increasing its quality and storage. Substances that can inhibit oxygen, absorb moisture, provide antimicrobial protection, control carbon dioxide, absorb ethylene, and neutralize undesirable flavors are in high demand [89,90]. Klein et al. developed stable antioxidant-antibacterial films via in situ bonds of silica nanoparticle precursors (SNP) with covalently linked curcumin or benzoic acid on polyvinyl alcohol (PVA). The designed films had exceptional antioxidant and antibacterial effects and improved hydrophobicity in protection against undesirable moisture. The growth of the *Listeria innocua* as a foodborne human pathogen was completely inhibited via PVA-SNP-ba films. The bacterium was reduced by 2.5 log using curcumin-loaded PVA-silica nanoparticles. Furthermore, the curcumin-loaded PVA-silica nanoparticles-based film displayed a 16 mM/g antioxidant effect as compared to 14.6 mM/g of PVA-silica nanoparticles-ba. Such an approach as the one described could be used as a scaffold for the fabrication of PVA-based packaging materials as an active alternative to silica. Thus, the safe entrapment of bioactive candidates with such biodegradable films could be valuable in both medicine and food industries [89].

**Figure 4:**
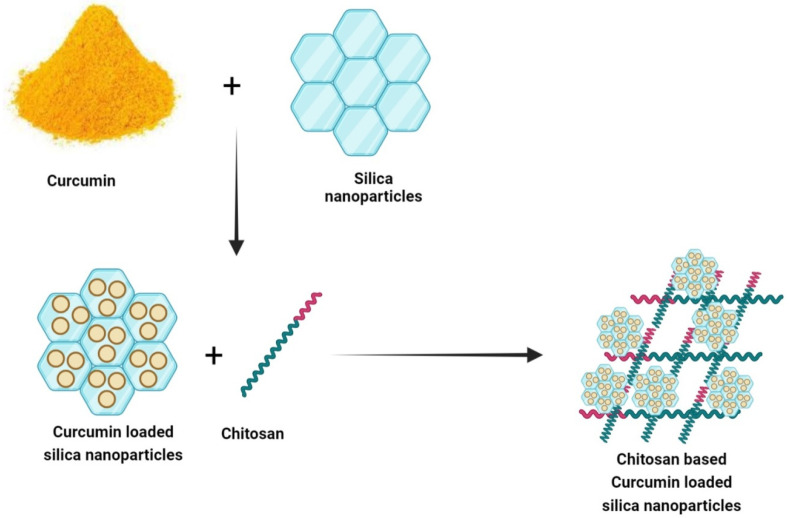
Preparation of CS/SBA-15/Cur bio-nanocomposite film.

In another study, to improve the functional properties of pure chitosan film, curcumin-loaded mesoporous silica nanoparticles (SBA-15) were inserted into chitosan (CS) film. Curcumin was loaded into SBA-15 (SBA-15-Cur) via the Rotator technique. The CS/SBA-15-Cur nanocomposite film was prepared by solvent casting (Figure 4). The SBA-15-Cur nanofiller was added to improve the mechanical properties of the nanocomposite film. The CS/SBA-15-Cur film showed effective antibacterial actions against *E. coli* and *S. aureus*. These results suggest that the CS/SBA-15-Cur nanocomposite film can be a promising material for food packaging [71].

## 9. Application of Silica Nanoparticles in the Detection of Bacterial Infections

Nanomaterials are widely utilized in the development and improvement of biosensor properties [91,92,93]. MSN detection has great potential for selective, sensitive, and rapid diagnosis of bacterial infections. While various investigations have focused on treating bacterial infections with MSN-based drug delivery formulas, aspects of diagnosis with MSNs have emerged recently. To date, there is no single diagnostic method that can meet the needs of the entire diagnostic procedure. Nevertheless, the MSN matrix with flexible design components offers improved and novel approaches for achieving selective, fast, and sensitive detection, as it can be isolated, identified, and implemented in-situ during imaging methods. The benefits of MSN-based drug delivery systems can be combined with diagnostic methods to provide MSN-based nano-transthetics to combat bacterial infection [94]. Huang et al. developed an acid-responsive microfluidic biosensor via ZnO-capped MSNs for signal amplification and curcumin as a signal reporter for rapid and sensitive *Salmonella* detection in fluorescent and colorimetric dual-modal sensing.

MSNs were initially incubated with curcumin to reach MSNs@CUR (MC NPs). It was then coated with ZnO nanoparticles to form MSNs@CUR@ZnO nanoparticles to inhibit the premature release of curcumin, followed by modification with polyclonal antibodies (pAbs) against *S. typhimurium* to immunize MSNs@CUR@ZnO@pAbs nanoparticles will be achieved. The *S. typhimurium* cells, the immune magnetic nanoparticles (MNPs), and the MSNs@CUR@ZnO@pAbs NPs were conjugated in the microfluidic chip with a Koch fractal mixing channel to form MNPs-bacteria-MSNs@CUR@ZnO@pAbs complexes. Lastly, acetic acid (HAc) was introduced to release curcumin from the complexes, and both the fluorescence and color changes were evaluated to determine the bacterial concentration. This designed biosensor was capable of quantitatively identifying *S. typhimurium*, ranging in 1.5 h from 10^2^ to 10^7^ CFU/mL [95].

## 10. Conclusions

In this study, the applications of silica nanoparticles with curcumin in the treatment of bacterial infections, food packaging, and the diagnosis of bacterial infections were investigated. These phytochemical silica nanoparticles with tremendous benefits—such as exceptional biocompatibility and cost-effective preparation—can be used as promising nanostructures to treat and diagnose infections. Because sufficient evidence is needed to prove the therapeutic efficacy and safety of MSNs, there is still a long way to go to fully realize MSN systems containing drugs in the clinical market.

## Figures and Tables

**Table 1:** Main characteristics of curcumin.

Curcumin		Ref
Chemical structure	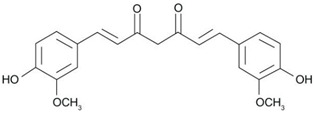 C21H20O6	[37]
	− curcumin can also be observed in different tautomeric forms.	
	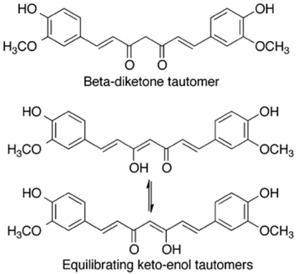	
	The functionalization of the aromatic rings can be performed by methoxy and hydroxy groups in an *ortho* position with respect to one another. The aromatic rings are linked to one another via a seven-carbon spacer that has two α, β-unsaturated carbonyl groups. Then, a beta-diketone and equilibrating keto-enol tautomeric forms of curcumin can be formed.	
Physicochemical possessions	− Hydrophobic affinity − Golden-yellow solid, with a molecular weight of 368 g mol^−1^ and a melting point of 183 °C	[37]
Sources	Turmeric (*Curcuma longa*)	[34,36,37]
Main pharmacological properties	− Anti-lipidemics, anti-diabetics, anti-tumor, anti-inflammatory, anti-fibrosis, anti-virus, anti-oxidation, and free radical scavengers	[38]
Toxic aspects	− Low toxicity, it is a non-toxic and safe material with a safe dose for human clinical trials larger than 120 mg/m^2^.	[39,40]
Bioavailability	Poor bioavailability	[41]
Pharmacokinetic issues	Effective first-pass metabolism and some degree of intestinal metabolism, mainly glucuronidation and sulfation of curcumin, might clarify its weak systemic accessibility once used through the oral way. A daily oral dose of 3.6 g of curcumin is well-matched with noticeable levels of the parent compound in colorectal tissue from patients with cancer.	[42]

**Table 2:** Methods to prepare the silica nanoparticles.

Synthesis Method	Description	Observation	Advantage	Disadvantage	Ref.
Sol–gel process	The method includes hydrolysis and condensation of metalalkoxides (Si(OR)4) or inorganic salts in the presence of mineral acid as a catalyst.	Mainly spherical nanoparticles, up to 200 nm depending on the reaction conditions.	Yields monodispersed particles with narrow size distribution	In mixing with water and ethanol, orthosilicate shows partial hydrolysis	[46,47,48,49]
Wet chemical synthesis	This method includes using tetraethyl orthosilicate, ethanol, water, and ammonium hydroxide with different surfactants.	Mainly amorphous nanoparticles in form and very fine in size	Very fine particle size	Long reaction time	[50]
Precipitation method	This method includes the precipitation of silica gel using sodium hydroxide, and sulfuric acid results.	Mainly spherical particles with 50 nm size.	High yield	Long reaction time	[51]
Acid treatment	This method includes the converting of the waste biomass combustion into silica nanoparticles.	Mainly amorphous, mono dispersed particles with 10 nm size.	Using of waste	Low yield	[52]

**Table 3:** Different types of MSNs

Type of Silica Particles	Pore Structure	Pore Volume (cm^3^/g)	Pore Size (nm)	Surface Area (m^2^/g)	Fields of Application	Ref.
Mobil Composition of Matter (MCM)-41	Hexagonal	0.5–1.5	1.5–10	800–1000	Drug delivery, catalytic applications, semiconductors, biofluids	[54,55]
MCM-48	Cubic	1.05	1.5–10	1000	Drug delivery, catalysis	[56,57]
Santa-Barbara Amorphous (SBA)-15	Hexagonal	0.50–0.65	5–8	400–800	Biosensors, drug delivery, catalysis	[58]
Folded sheets mesoporous materials (FSM)-16	Honeycomb	0.28–0.83	1.5–4	680–1000	Super high-speed light switching devices, pharmaceutical	[59,60]
Technische Universiteit Delft (TUD)-1	Foam-like	0.5–1.7	4–18	400–1000	Drug delivery, catalysis	[61,62]
Sylysia350	Disordered	1.6	21	300	Pharmaceutical, catalyst supports, filters	[63,64]
Syloid244	Disordered	1.42	19	311	Drug delivery, catalysis	[65]

## Data Availability

Not applicable.

## References

[1] Li Z., Barnes J.C., Bosoy A., Stoddart J.F., Zink J.I. (2012). Mesoporous silica nanoparticles in biomedical applications. Chem. Soc. Rev..

[2] Kankala R.K., Han Y., Na J., Lee C., Sun Z., Wang S., Kimura T., Ok Y.S., Yamauchi Y., Chen A. (2020). Nanoarchitectured structure and surface biofunctionality of mesoporous silica nanoparticles. Adv. Mater..

[3] Zhou Y., Quan G., Wu Q., Zhang X., Niu B., Wu B., Hunag Y., Pan X., Wu C. (2018). Mesoporous silica nanoparticles for drug and gene delivery. Acta Pharm. Sin. B.

[4] Vallet-Regí M., Colilla M., Izquierdo-Barba I., Manzano M. (2017). Mesoporous silica nanoparticles for drug delivery: Current insights. Molecules.

[5] Frickenstein A., Hagood J., Britten C., Abbott B., McNally M., Vopat C., Patterson E., MacCuaig W., Jain A., Walters K. (2021). Mesoporous silica nanoparticles: Properties and strategies for enhancing clinical effect. Pharmaceutics.

[6] Zhang Y., Wang J., Bai X., Jiang T., Zhang Q., Wang S. (2012). Mesoporous silica nanoparticles for increasing the oral bioavailability and permeation of poorly water soluble drugs. Mol. Pharm..

[7] Alyassin Y., Sayed E.G., Mehta P., Ruparelia K., Arshad M.S., Rasekh M., Shepherd J., Kucuk I., Wilson P.B., Singh N. (2020). Application of mesoporous silica nanoparticles as drug delivery carriers for chemotherapeutic agents. Drug Discov. Today.

[8] Kim K.M., Kim H.M., Lee W.J., Lee C.W., Kim T.I., Lee J.K., Jeong J., Paek S.M., Oh J.M. (2014). Surface treatment of silica nanoparticles for stable and charge-controlled colloidal silica. Int. J. Nanomed..

[9] Nel A.E., Mädler L., Velegol D., Xia T., Hoek E.M.V., Somasundaran P., Klaessig F., Castranova V., Thompson M. (2009). Understanding biophysicochemical interactions at the nano–bio interface. Nat. Mater..

[10] Bansal S.S., Goel M., Aqil F., Vadhanam M.V., Gupta R.C. (2011). Advanced drug delivery systems of curcumin for cancer chemoprevention. Cancer Prev. Res..

[11] Khezri K., Dizaj S.M., Saadat Y.R., Sharifi S., Shahi S., Ahmadian E., Eftekhari A., Abdolahinia E.D., Lotfipour F. (2021). Osteogenic Differentiation of Mesenchymal Stem Cells via Curcumin-Containing Nanoscaffolds. Stem Cells Int..

[12] Sharifi S., Fathi N., Memar M.Y., Khatibi S.M.H., Khalilov R., Negahdari R., Vahed S.Z., Maleki Dizaj S. (2020). Anti-microbial activity of curcumin nanoformulations: New trends and future perspectives. Phytother. Res..

[13] Ghavimi M.A., Shahabadi A.B., Jarolmasjed S., Memar M.Y., Dizaj S.M., Sharifi S. (2020). Nanofibrous asymmetric collagen/curcumin membrane containing aspirin-loaded PLGA nanoparticles for guided bone regeneration. Sci. Rep..

[14] Sharifi S., Vahed S.Z., Ahmadian E., Dizaj S.M., Abedi A., Khatibi S.M.H., Samiei M. (2019). Stem Cell Therapy: Curcumin Does the Trick. Phytother. Res..

[15] Samiei M., Abedi A., Sharifi S., Dizaj S.M. (2021). Early Osteogenic Differentiation Stimulation of Dental Pulp Stem Cells by Calcitriol and Curcumin. Stem Cells Int..

[16] Mishra B., Patel B.B., Tiwari S. (2010). Colloidal nanocarriers: A review on formulation technology, types and applications toward targeted drug delivery. Nanomed. Nanotechnol. Biol. Med..

[17] Oerlemans C., Bult W., Bos M., Storm G., Nijsen J.F.W., Hennink W.E. (2010). Polymeric Micelles in Anticancer Therapy: Targeting, Imaging and Triggered Release. Pharm. Res..

[18] Bohlouli S., Gharehbagh F.J., Abdolahinia E.D., Kouhsoltani M., Ebrahimi G., Roshangar L., Imani A., Sharifi S., Dizaj S.M. (2021). Preparation, Characterization, and Evaluation of Rutin Nanocrystals as an Anticancer Agent against Head and Neck Squamous Cell Carcinoma Cell Line. J. Nanomater..

[19] Aqil F., Munagala R., Jeyabalan J., Vadhanam M.V. (2013). Bioavailability of phytochemicals and its enhancement by drug delivery systems. Cancer Lett..

[20] Negahdari R., Bohlouli S., Sharifi S., Dizaj S.M., Saadat Y.R., Khezri K., Jafari S., Ahmadian E., Jahandizi N.G., Raeesi S. (2021). Therapeutic benefits of rutin and its nanoformulations. Phytother. Res..

[21] Jafari S., Derakhshankhah H., Alaei L., Fattahi A., Varnamkhasti B.S., Saboury A.A. (2019). Mesoporous silica nanoparticles for therapeutic/diagnostic applications. Biomed. Pharmacother..

[22] Memar M.Y., Yekani M., Ghanbari H., Nabizadeh E., Vahed S.Z., Dizaj S.M., Sharifi S. (2021). Antimicrobial and antibiofilm activities of meropenem loaded-mesoporous silica nanoparticles against carbapenem-resistant Pseudomonas aeruginosa. J. Biomater. Appl..

[23] Memar M.Y., Yekani M., Ghanbari H., Shahi S., Sharifi S., Dizaj S.M. (2020). Biocompatibility, cytotoxicity and antibacterial effects of meropenem-loaded mesoporous silica nanoparticles against carbapenem-resistant Enterobacteriaceae. Artif. Cells Nanomed. Biotechnol..

[24] Slowing I., Trewyn B.G., Lin V.S.-Y. (2006). Effect of Surface Functionalization of MCM-41-Type Mesoporous Silica Nanoparticles on the Endocytosis by Human Cancer Cells. J. Am. Chem. Soc..

[25] Chung T.-H., Wu S.-H., Yao M., Lu C.-W., Lin Y.-S., Hung Y., Mou C.-Y., Chen Y.-C., Huang D.-M. (2007). The effect of surface charge on the uptake and biological function of mesoporous silica nanoparticles in 3T3-L1 cells and human mesenchymal stem cells. Biomaterials.

[26] Armat M., Bakhshaiesh T.O., Sabzichi M., Shanehbandi D., Sharifi S., Molavi O., Mohammadian J., Hejazi M.S., Samadi N. (2016). The role of Six1 signaling in paclitaxel-dependent apoptosis in MCF-7 cell line. Bosn. J. Basic Med Sci..

[27] Bakhshaiesh T.O., Armat M., Shanehbandi D., Sharifi S., Baradaran B., Hejazi M.S., Samadi N. (2015). Arsenic Trioxide Promotes Paclitaxel Cytotoxicity in Resistant Breast Cancer Cells. Asian Pac. J. Cancer Prev..

[28] Mohseni M., Samadi N., Ghanbari P., Yousefi B., Tabasinezhad M., Sharifi S., Nazemiyeh H. (2016). Co-treatment by docetaxel and vinblastine breaks down P-glycoprotein mediated chemo-resistance. Iran. J. Basic Med. Sci..

[29] Vuong T.V. (2021). Natural Products and Their Derivatives with Antibacterial, Antioxidant and Anticancer Activities. Antibiotics.

[30] Gupta S.C., Kismali G., Aggarwal B.B. (2013). Curcumin, a component of turmeric: From farm to pharmacy. BioFactors.

[31] Dizaj S.M., Alipour M., Abdolahinia E.D., Ahmadian E., Eftekhari A., Forouhandeh H., Saadat Y.R., Sharifi S., Vahed S.Z. (2022). Curcumin nanoformulations: Beneficial nanomedicine against cancer. Phytother. Res..

[32] Sharifi S., Khosroshahi A.Z., Dizaj S.M., Rezaei Y. (2021). Preparation, Physicochemical Assessment and the Antimicrobial Action of Hydroxyapatite–Gelatin/Curcumin Nanofibrous Composites as a Dental Biomaterial. Biomimetics.

[33] Negahdari R., Ghavimi M.A., Barzegar A., Memar M.Y., Balazadeh L., Bohlouli S., Sharifi S., Dizaj S.M. (2021). Antibacterial effect of nanocurcumin inside the implant fixture: An in vitro study. Clin. Exp. Dent. Res..

[34] Panahi Y., Hosseini M.S., Khalili N., Naimi E., Majeed M., Sahebkar A. (2015). Antioxidant and anti-inflammatory effects of curcuminoid-piperine combination in subjects with metabolic syndrome: A randomized controlled trial and an updated meta-analysis. Clin. Nutr..

[35] Hewlings S.J., Kalman D.S. (2017). Curcumin: A review of its effects on human health. Foods.

[36] Pant P., Gupta C., Kumar S., Grewal A., Garg S., Rai A. (2020). Curcumin loaded Silica Nanoparticles and their therapeutic applications: A review. J. Mater. NanoSci..

[37] Payton F., Sandusky P., Alworth W.L. (2007). NMR Study of the Solution Structure of Curcumin. J. Nat. Prod..

[38] Fu Y.-S., Chen T.-H., Weng L., Huang L., Lai D., Weng C.-F. (2021). Pharmacological properties and underlying mechanisms of curcumin and prospects in medicinal potential. Biomed. Pharmacother..

[39] Soleimani V., Sahebkar A., Hosseinzadeh H. (2018). Turmeric (Curcuma longa) and its major constituent (curcumin) as nontoxic and safe substances: Review. Phyther. Res..

[40] Storka A., Vcelar B., Klickovic U., Gouya G., Weisshaar S., Aschauer S., Bolger G., Helson L., Wolzt M. (2015). Safety, tolerability and pharmacokinetics of liposomal curcumin (Lipocurc™) in healthy humans. Int. J. Clin. Pharmacol. Ther..

[41] Lopresti A.L. (2018). The problem of curcumin and its bioavailability: Could its gastrointestinal influence contribute to its overall health-enhancing effects?. Adv. Nutr..

[42] Sharma R., Steward W., Gescher A. (2007). The molecular targets and therapeutic uses of curcumin in health and disease. Adv. Exp. Med. Biol..

[43] Selvarajan V., Obuobi S.A.O., Ee P.L.R. (2020). Silica Nanoparticles—A Versatile Tool for the Treatment of Bacterial Infections. Front. Chem..

[44] Holkar C.R., Jadhav A.J., Karekar S.E., Pandit A.B., Pinjari D.V. (2016). Recent developments in synthesis of nanomaterials utilized in polymer based composites for food packaging applications. J. Food Bioeng. Nanoprocess.

[45] Waqas H., Khan T.A., Hameed A., Abbasi R., Naz S., Ahmed M.J., Shah Z.H., Hassan S.M., Qureshi A.H., Ahmed M.B. (2020). In vitro cytotoxicity study of virgin, ethylenediaminetetraacetic acid-and hexamethylenetetramine-capped silica particles synthesized by precipitation method. Chem. Pap..

[46] Hassan A., Abdelghny A.M., Elhadidy H., Youssef A.M. (2013). Synthesis and characterization of high surface area nanosilica from rice husk ash by surfactant-free sol–gel method. J. Sol-Gel Sci. Technol..

[47] Le V.H., Thuc C.N.H., Thuc H.H. (2013). Synthesis of silica nanoparticles from Vietnamese rice husk by sol–gel method. Nanoscale Res. Lett..

[48] Rahman I.A., Padavettan V. (2012). Synthesis of silica nanoparticles by sol-gel: Size-dependent properties, surface modification, and applications in silica-polymer nanocomposites—A review. J. Nanomater..

[49] Singh L.P., Bhattacharyya S.K., Kumar R., Mishra G., Sharma U., Singh G., Ahalawat S. (2014). Sol-Gel processing of silica nanoparticles and their applications. Adv. Colloid Interface Sci..

[50] Stanley R., Nesaraj A.S. (2014). Effect of surfactants on the wet chemical synthesis of silica nanoparticles. Int. J. Appl. Sci. Eng..

[51] Jal P., Sudarshan M., Saha A., Patel S., Mishra B. (2004). Synthesis and characterization of nanosilica prepared by precipitation method. Colloids Surf. A Physicochem. Eng. Asp..

[52] Rani A., Sanal S., Jacob T., Jacob G., Desy P.K., Manivarnan N.K. (2014). Silica nano particles synthesized from boiler spent ash: Value addition to an industrial waste. Chem. Mater. Res..

[53] McCarthy C.A., Ahern R.J., Dontireddy R., Ryan K.B., Crean A.M. (2016). Mesoporous silica formulation strategies for drug dissolution enhancement: A review. Expert. Opin. Drug. Deliv..

[54] Bhattacharyya S., Lelong G., Saboungi M.-L. (2006). Recent progress in the synthesis and selected applications of MCM-41: A short review. J. Exp. Nanosci..

[55] Sharifi S., Dalir Abdolahinia E., Maleki Dizaj S., Nejatian T., Seydi Z., Kouhsoltani M. (2022). Preparation, the physicochemical assessment, and the cytotoxicity of Cisplatin-loaded mesoporous Silica nanoparticles against head and neck squamous cell carcinoma cell line. Int. J. Nano Dimens..

[56] Aghaei H., Nourbakhsh A.A., Karbasi S., Javad Kalbasi R., Rafienia M., Nourbakhsh N., Bonakdar S., Mackenzie K.J.D. (2014). Investigation on bioactivity and cytotoxicity of mesoporous nano-composite MCM-48/hydroxyapatite for ibuprofen drug delivery. Ceram. Int..

[57] Schumacher K., Grün M., Unger K. (1999). Novel synthesis of spherical MCM-48. Microporous Mesoporous Mater..

[58] Lee J.W., Cho D.L., Shim W.G., Moon H. (2004). Application of mesoporous MCM-48 and SBA-15 materials for the separation of biochemicals dissolved in aqueous solution. Korean J. Chem. Eng..

[59] Wakayama H., Fukushima Y. (2009). Preparation of Nanoparticles in Nanoporous Silica, FSM-16. J. Chem. Eng. Jpn..

[60] Tozuka Y., Wongmekiat A., Kimura K., Moribe K., Yamamura S., Yamamoto K. (2005). Effect of Pore Size of FSM-16 on the Entrapment of Flurbiprofen in Mesoporous Structures. Chem. Pharm. Bull..

[61] Hamdy M. (2005). Functionalized TUD-1: Synthesis, Characterization and (Photo-) Catalytic Performance.

[62] Heikkilä T., Salonen J., Tuura J., Hamdy M.S., Mul G., Kumar N., Salmi T., Murzin D., Laitinen L., Kaukonen A. (2007). Mesoporous silica material TUD-1 as a drug delivery system. Int. J. Pharm..

[63] Ahuja G., Pathak K. (2009). Porous carriers for controlled/modulated drug delivery. Indian J. Pharm. Sci..

[64] Planinšek O., Kovačič B., Vrečer F. (2011). Carvedilol dissolution improvement by preparation of solid dispersions with porous silica. Int. J. Pharm..

[65] Krysztafkiewicz A., Binkowski S., Wysocka I. (2003). Pigments on amorphous silica carriers. Powder Technol..

[66] Álvarez E., González B., Lozano D., Doadrio A.L., Colilla M., Izquierdo-Barba I. (2021). Nanoantibiotics Based in Mesoporous Silica Nanoparticles: New Formulations for Bacterial Infection Treatment. Pharmaceutics.

[67] Aguilera-Correa J., Gisbert-Garzarán M., Mediero A., Carias-Cálix R., Jiménez-Jiménez C., Esteban J., Vallet-Regí M. (2022). Arabic gum plus colistin coated moxifloxacin-loaded nanoparticles for the treatment of bone infection caused by Escherichia coli. Acta Biomater..

[68] Song Y., Cai L., Tian Z., Wu Y., Chen J. (2020). Phytochemical Curcumin-Coformulated, Silver-Decorated Melanin-like Polydopamine/Mesoporous Silica Composites with Improved Antibacterial and Chemotherapeutic Effects against Drug-Resistant Cancer Cells. ACS Omega.

[69] Cheng Y., Zhang Y., Deng W., Hu J. (2020). Antibacterial and anticancer activities of asymmetric lollipop-like mesoporous silica nanoparticles loaded with curcumin and gentamicin sulfate. Colloids Surf. B Biointerfaces.

[70] Kuthati Y., Kankala R.K., Busa P., Lin S.-X., Deng J.-P., Mou C.-Y., Lee C.-H. (2017). Phototherapeutic spectrum expansion through synergistic effect of mesoporous silica trio-nanohybrids against antibiotic-resistant gram-negative bacterium. J. Photochem. Photobiol. B Biol..

[71] Wu C., Zhu Y., Wu T., Wang L., Yuan Y., Chen J., Hu Y., Pang J. (2019). Enhanced functional properties of biopolymer film incorporated with curcurmin-loaded mesoporous silica nanoparticles for food packaging. Food Chem..

[72] Li D., Nie W., Chen L., Miao Y., Zhang X., Chen F., Yu B., Ao R., Yu B., He C. (2017). Fabrication of curcumin-loaded mesoporous silica incorporated polyvinyl pyrrolidone nanofibers for rapid hemostasis and antibacterial treatment. RSC Adv..

[73] Barchitta M., Maugeri A., Favara G., Lio R.M.S., Evola G., Agodi A., Basile G. (2019). Nutrition and Wound Healing: An Overview Focusing on the Beneficial Effects of Curcumin. Int. J. Mol. Sci..

[74] Hamam F., Nasr A. (2020). Curcumin-loaded mesoporous silica particles as wound-healing agent: An In vivo study. Saudi J. Med. Med Sci..

[75] Mirzahosseinipour M., Khorsandi K., Hosseinzadeh R., Ghazaeian M., Shahidi F.K. (2020). Antimicrobial photodynamic and wound healing activity of curcumin encapsulated in silica nanoparticles. Photodiagn. Photodyn. Ther..

[76] Xi Y., Ge J., Wang M., Chen M., Niu W., Cheng W., Xue Y., Lin C., Lei B. (2020). Bioactive Anti-inflammatory, Antibacterial, Antioxidative Silicon-Based Nanofibrous Dressing Enables Cutaneous Tumor Photothermo-Chemo Therapy and Infection-Induced Wound Healing. ACS Nano.

[77] Rathinavel S., Korrapati P.S., Kalaiselvi P., Dharmalingam S. (2021). Mesoporous silica incorporated PCL/Curcumin nanofiber for wound healing application. Eur. J. Pharm. Sci..

[78] Rathinavel S., Ekambaram S., Korrapati P.S., Sangeetha D. (2020). Design and fabrication of electrospun SBA-15-incorporated PVA with curcumin: A biomimetic nanoscaffold for skin tissue engineering. Biomed. Mater..

[79] Steinmann J., Buer J., Pietschmann T., Steinmann E. (2013). Anti-infective properties of epigallocatechin-3-gallate (EGCG), a component of green tea. Br. J. Pharmacol..

[80] Connell B.J., Chang S.-Y., Prakash E., Yousfi R., Mohan V., Posch W., Wilflingseder D., Moog C., Kodama E.N., Clayette P. (2016). A Cinnamon-Derived Procyanidin Compound Displays Anti-HIV-1 Activity by Blocking Heparan Sulfate- and Co-Receptor- Binding Sites on gp120 and Reverses T Cell Exhaustion via Impeding Tim-3 and PD-1 Upregulation. PLoS ONE.

[81] Mounce B.C., Cesaro T., Carrau L., Vallet T., Vignuzzi M. (2017). Curcumin inhibits Zika and chikungunya virus infection by inhibiting cell binding. Antivir. Res..

[82] Praditya D., Kirchhoff L., Brüning J., Rachmawati H., Steinmann J., Steinmann E. (2019). Anti-infective Properties of the Golden Spice Curcumin. Front. Microbiol..

[83] Kutluay S.B., Doroghazi J., Roemer M.E., Triezenberg S.J. (2008). Curcumin inhibits herpes simplex virus immediate-early gene expression by a mechanism independent of p300/CBP histone acetyltransferase activity. Virology.

[84] Maher D.M., Bell M.C., O’Donnell E.A., Gupta B.K., Jaggi M., Chauhan S.C. (2011). Curcumin suppresses human papillomavirus oncoproteins, restores p53, rb, and ptpn13 proteins and inhibits benzo [a] pyrene-induced upregulation of HPV E7. Mol. Carcinog..

[85] Sharma A., Yadav A., Gupta N., Sharma S., Kakkar R., Cwiklinski K., Quaye E., Mahajan S.D., Schwartz S.A., Sharma R.K. (2019). Multifunctional mesoporous curcumin encapsulated iron-phenanthroline nanocluster: A new Anti-HIV agent. Colloids B Biointerfaces.

[86] Lo T.-H., Wu Z.Y., Chen S.Y., Meng F.Y., Chou P.T., Wang C.M., Lin H.M. (2021). Curcumin-loaded mesoporous silica nanoparticles with dual-imaging and temperature control inhibits the infection of Zika virus. Microporous Mesoporous Mater..

[87] De La Franier B., Asker D., Berg D.v.D., Hatton B., Thompson M. (2021). Reduction of microbial adhesion on polyurethane by a sub-nanometer covalently-attached surface modifier. Colloids Surf. B Biointerfaces.

[88] Han J.-W., Ruiz-Garcia L., Qian J.-P., Yang X.-T. (2018). Food Packaging: A Comprehensive Review and Future Trends. Compr. Rev. Food Sci. Food Saf..

[89] Klein M., Filossof A.M., Ashur I., Vernick S., Natan-Warhaftig M., Rodov V., Banin E., Poverenov E. (2021). In Situ Grafting of Silica Nanoparticle Precursors with Covalently Attached Bioactive Agents to Form PVA-Based Materials for Sustainable Active Packaging. Polymers.

[90] Gaikwad K.K., Singh S., Negi Y.S. (2020). Ethylene scavengers for active packaging of fresh food produce. Environ. Chem. Lett..

[91] Hamidi-Asl E., Raoof J.B., Hejazi M.S., Sharifi S., Golabi S.M., Palchetti I., Mascini M. (2015). A Genosensor for Point Mutation Detection of P53 Gene PCR Product Using Magnetic Particles. Electroanalysis.

[92] Ahmadian E., Dizaj S.M., Sharifi S., Shahi S., Khalilov R., Eftekhari A., Hasanzadeh M. (2019). The potential of nanomaterials in theranostics of oral squamous cell carcinoma: Recent progress. TrAC Trends Anal. Chem..

[93] Eftekhari A., Ahmadian E., Salatin S., Sharifi S., Dizaj S.M., Khalilov R., Hasanzadeh M. (2019). Current analytical approaches in diagnosis of melanoma. TrAC Trends Anal. Chem..

[94] Karaman D., Pamukçu A., Karakaplan M.B., Kocaoglu O., Rosenholm J.M. (2021). Recent Advances in the Use of Mesoporous Silica Nanoparticles for the Diagnosis of Bacterial Infections. Int. J. Nanomed..

[95] Huang F., Guo R., Xue L., Cai G., Wang S., Li Y., Lin J. (2020). An Acid-Responsive microfluidic salmonella biosensor using curcumin as signal reporter and ZnO-capped mesoporous silica nanoparticles for signal amplification. Sens. Actuators B Chem..

